# Distinct risk factors of lateral lymph node metastasis in patients with papillary thyroid cancer based on age stratification

**DOI:** 10.1186/s12893-024-02309-2

**Published:** 2024-01-13

**Authors:** Huizhu Cai, Lingdun Zhuge, Zehao Huang, Shixu Wang, Ping Shi, Dangui Yan, Lijuan Niu, Zhengjiang Li

**Affiliations:** 1 Department of Head and Neck Surgery, National Cancer Center/National Clinical Research Center for Cancer/Cancer Hospital, Chinese Academy of Medical Sciences and Peking Union Medical College, Beijing, China; 2 Department of ENT, The Fourth Affiliated Hospital of Hebei Medical University, Shijiazhuang, China; 3 Department of Ultrasound, National Cancer Center/National Clinical Research Center for Cancer/Cancer Hospital, Chinese Academy of Medical Sciences and Peking Union Medical College, Beijing, China

**Keywords:** Papillary thyroid carcinoma, Lymph node metastasis, Neck dissection, Predict model, Age stratification

## Abstract

**Introduction:**

Studies have revealed that age is associated with the risk of lateral lymph node metastasis (LLNM) in papillary thyroid cancer (PTC). This study aimed to identify the optimal cut point of age for a more precise prediction model of LLNM and to reveal differences in risk factors between patients of distinct age stages.

**Methods:**

A total of 499 patients who had undergone thyroidectomy and lateral neck dissection (LND) for PTC were enrolled. The locally weighted scatterplot smoothing (LOWESS) curve and the ‘changepoint’ package were used to identify the optimal age cut point using R. Multivariate logistic regression analysis was performed to identify independent risk factors of LLNM in each group divided by age.

**Results:**

Younger patients were more likely to have LLNM, and the optimal cut points of age to stratify the risk of LLNM were 30 and 45 years old. Central lymph node metastasis (CLNM) was a prominent risk factor for further LNM in all patients. Apart from CLNM, sex(*p* = 0.033), tumor size(*p* = 0.027), and tumor location(*p* = 0.020) were independent predictors for patients younger than 30 years old; tumor location(*p* = 0.013), extra-thyroidal extension(*p* < 0.001), and extra-nodal extension(*p* = 0.042) were independent risk factors for patients older than 45 years old.

**Conclusions:**

Our study could be interpreted as an implication for a change in surgical management. LND should be more actively performed when CLNM is confirmed; for younger patients with tumors in the upper lobe and older patients with extra-thyroidal extension tumors, more aggressive detection of the lateral neck might be considered.

## Background

The incidence of thyroid mass has rapidly increased in the past few decades [1, 2]. Papillary thyroid carcinoma (PTC), the most common type of thyroid malignancy, is prone to lymph node metastasis (LNM). As it is acknowledged that PTC has quite a low mortality rate and LNM has a less significant factor influencing survival rate [3], several recent studies found that LNM still negatively affected long-term recurrence [4–6]. The most common site of LNM in PTC is the central compartment, followed by the lateral compartment.

According to the latest American Thyroid Association (ATA) guidelines and National Comprehensive Cancer Network (NCCN) guidelines, lateral neck dissection (LND) is only recommended for patients who were diagnosed with lateral lymph node metastasis (LLNM) before or during the operation. Prophylactic LND is not suggested. However, the sensitivity of evaluations, including preoperative imaging examination and intraoperative frozen pathology, is limited [7, 8], and occult LNM may lead to relapse and secondary operation. Previous studies reported that the incidence of occult LLNM in PTC patients could reach 18.6-64% [9–11]. Thus, constructing a precise predictive model for LLNM seems essential in improving the efficacy of operations.

It is proved that age is one of the most important prognostic factors of overall survival and disease-specific survival in PTC [12–14]. Several studies have also reported that the risk of LLNM decreased as age increased simultaneously [15–17]. However, the exact tendency of the risk ratio of LLNM as age changes is still unclear. Therefore, the cut point of patient age to stratify the risk of LLNM has not been well established yet. In addition, studies focusing on investigating distinct predictors of LLNM according to patient age were limited, leading to a lack of evidence to support more specific surgical strategies for PTC patients of different ages. As the risk of LLNM changes with age, a novel prediction model based on age stratification might be more accurate and benefit clinical practice.

Our study aimed to identify the differences in clinicopathological characteristics, especially age, between patients with or without LLNM, to determine the optimal breakpoint of age for a more precise prediction model of LLNM, and to reveal differences in risk factors between patients of distinct age stages.

## Materials and methods

### Patients

This institutional ethics committee-approved retrospective study was conducted by searching the Department of Head and Neck Surgery, Cancer Hospital of Chinese Academy of Medical Sciences, and Peking Union Medical College databases from February 2016 to January 2020. Patients were included if they met all the following inclusion criteria: (i) primary thyroid lesion was confirmed PTC by pathology; (ii) patients had undergone thyroid lobectomy or total thyroidectomy and LND by the same experienced clinician; (iii) patients had no history of neck surgery, or radioactive treatment; (iv) patients had no history of other systematic malignant tumors. Clinical data were collected on demographics (age, gender, etc.) and tumor characteristics (tumor size, tumor location, multifocality, etc.). Informed consent was obtained from all participants. We explained intraoperative and postoperative risks to all patients in detail before surgery to ensure they fully understood the disease and surgical methods.

### Surgery

We performed total thyroidectomy on patients with bilateral PTC. For unilateral PTC patients, total thyroidectomy or lobectomy plus isthmusectomy were performed depending on patients’ wishes. When unilateral PTC patients met one of the following conditions, total thyroidectomy was recommended: tumor size > 4 cm, multifocality in one lobe, contralateral benign nodules, or distant metastasis (according to guidelines of the Chinese Thyroid Association).

All patients received central lymph node dissection. LND of level II-IV or II-V was performed on patients pathologically confirmed to have LLNM by fine-needle aspiration or intraoperative frozen biopsy in the lateral compartments. Additionally, when LNM in the central compartment was found before or during surgery, we performed LND of level III and IV based on the original thyroid collar incision. After surgeries, all specimens had pathologically examinations for LNM diagnosing.

### Statistical analysis

All statistical analyses were performed using SPSS version 26.0 (IBM Inc, Armonk, NY, USA) and R version 4.2.1(www.r-project.org). Measurement data were expressed in mean ± standard deviation, and independent samples t-test was used for comparative analysis; categorical variables were expressed as numbers [percent (%)] and were compared by the chi-square test. Variables with *p* value less than 0.05 in univariate analysis were included in multivariate logistic regression analysis to identify independent risk factors. *P* < 0.05 was considered statistically significant.

The impact of age on LLNM was evaluated by logistic regression analysis. A locally weighted scatterplot smoothing (LOWESS) curve was used to fit and visualize the tendency of the OR of LLNM according to age. The structural break point of the fitting curves was considered as the optimal cutoff point and was determined by ‘changepoint’ package using R.

## Results

### Patient characteristics

A total of 499 patients were enrolled in this study. Among them, 396 (88.2%) were confirmed to have LLNM, and 103(22.9%) were absent from LLNM. A total of 163 patients (32.7%) undergone prophylactic lateral neck dissection of level III and IV as LNM in the central compartment was found, and 36.8% of these patients were confirmed to have occult LLNM. The details of characteristics were presented in Table 1, which showed statistical differences between the two groups with regard to age(*p* = 0.003), gender(*p* = 0.005), tumor size(*p* = 0.009), tumor location(*p* = 0.011), multifocality(*p* < 0.001), bilaterality(*p* = 0.002), extra-thyroidal extension(*p* < 0.001), extra-nodal extension(*p* < 0.001), and central lymph node metastasis (CLNM) (*p* < 0.001). No statistically significant difference between the two groups was observed in thyroiditis.

**Table 1:** Clinicopathologic characteristics of patients

		LLNM		
Characteristic	Overall(*n* = 499)	Present(*n* = 396)	Absent(*n* = 103)	*p* value
**Age(years)**				**0.003**
Mean (SD)	39.0(11.7)	38.2(11.4)	42.1(12.4)	
**Gender,n(%)**				**0.005**
Male	159(31.9)	138(34.8)	21(20.4)	
Female	340(68.1)	258(65.2)	82(79.6)	
**Tumor size,n(%)**				**0.009**
≥ 20 mm	105(21.0)	93(23.5)	12(11.7)	
< 20 mm	394(79.0)	303(76.5)	91(88.3)	
**Location,n(%)**				**0.011**
Upper lobe	142(28.5)	123(31.1)	19(18.4)	
Others	357(71.5)	273(68.9)	84(81.6)	
**Thyroiditis,n(%)**				0.521
Present	209(41.9)	163(41.2)	46(44.7)	
Absent	290(58.1)	233(58.8)	57(55.3)	
**Multifocality,n(%)**				**< 0.001**
Present	305(61.1)	262(66.2)	43(41.7)	
Absent	194(38.9)	134(33.8)	60(58.3)	
**Bilaterality,n(%)**				**0.002**
Present	232(46.5)	198(50.0)	34(33.0)	
Absent	267(53.5)	198(50.0)	69(67.0)	
**Extra-thyroidal extension,n(%)**				**< 0.001**
Present	391(78.4)	325(82.1)	66(64.1)	
Absent	108(21.6)	71(17.9)	37(35.9)	
**Extra-nodal extension,n(%)**				**< 0.001**
Present	156(31.3)	144(36.4)	12(11.7)	
Absent	343(68.7)	252(63.6)	91(88.3)	
**CLNM,n(%)**				**< 0.001**
Present	392(78.6)	339(85.6)	53(51.5)	
Absent	107(21.4)	57(14.4)	50(48.5)	

### Predictive value of age for LLNM

**Fig. 1:**
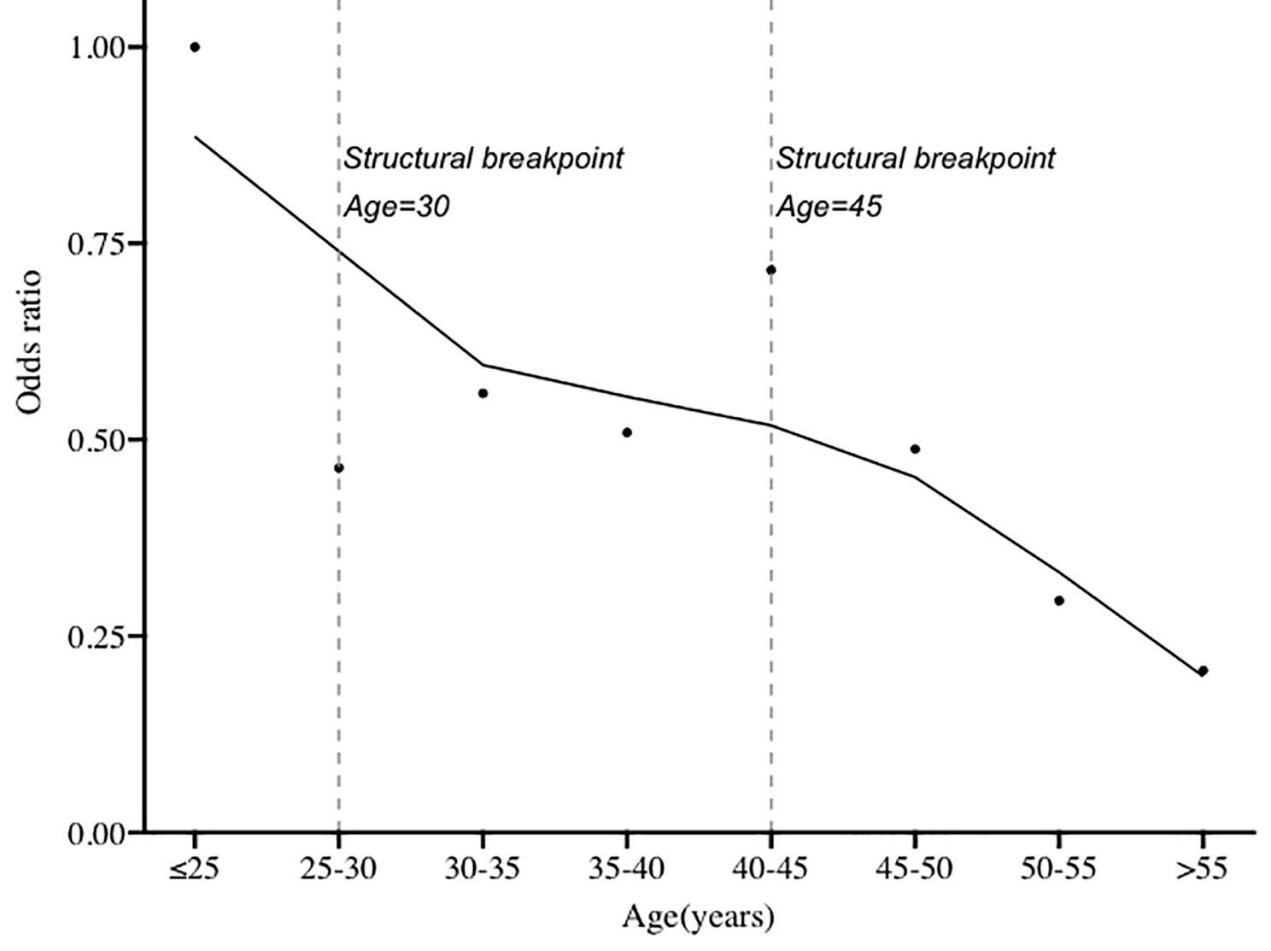
Associations of age with odds ratio for lateral lymph node metastasis. The fitting curve was determined by the locally weighted scatterplot smoothing (LOWESS), and structural breakpoints were identified by the ‘changepoint’ package in R

A logistic regression analysis was performed to identify further the relationship between age and LLNM (Table 2). The results indicated that age was significantly related to LLNM (*p* = 0.043). As shown in Fig. 1, the LOWESS curve fitting the trend of odds ratio as above demonstrated that the risk of LLNM decreased as age grew. Correspondingly, structural breakpoints of the fitting curve identified by R using the ‘changepoint’ package confirmed that optimal age cut points were 30 and 45.

**Table 2:** Odds ratio of lateral lymph node metastasis of different age

	OR	95%CI		*p* value
		Lower	Upper	
**Age(years)**				**0.043**
≤ 25	1.000			
25–30	0.464	0.165	1.305	0.146
30–35	0.559	0.206	1.517	0.253
35–40	0.509	0.182	1.426	0.199
40–45	0.716	0.247	2.073	0.538
45–50	0.488	0.163	1.460	0.199
50–55	0.295	0.103	0.845	0.023
> 55	0.206	0.073	0.583	0.003

Patients were divided into three groups based on cut points of 30 and 45 years old to verify the predictive value of age. The likelihood of LLNM was significantly different between the three groups, and older patients tended to have a lower risk of LLNM. After multivariate analysis, the risk of LLNM in the patient groups divided using the identified cut points remained distinctly different (*p* = 0.020) (Table 3). P for interaction was also calculated between age group and other risk factors of LLNM, apart from extra-thyroidal extension, which showed an interaction p-value of 0.011 with age, there was no statistically significant interaction between age group and other risk factors.

**Table 3:** Univariate and multivariate logistic regression analysis of characteristics associated with lateral lymph node metastasis

	Univariate analysis				Multivariate analysis		
Characteristic	OR	95% CI	*p* value		OR	95% CI	*p* value
**Age(years)**			**0.015**				**0.020**
> 45	0.488	0.267–0.891	0.019		0.030	0.206–0.921	0.030
30–45	0.933	0.520–1.674	0.817		0.999	0.511–1.952	0.998
≤ 30	1.000				1.000		
**Gender**			**0.006**				**0.030**
Male	2.089	1.239–3.520			1.916	1.064–3.452	
Female	1.000				1.000		
**Tumor size**			**0.010**				**0.002**
≥ 20 mm	2.328	1.221–4.437			3.212	1.535–6.822	
< 20 mm	1.000				1.000		
**Location**			**0.013**				**< 0.001**
Upper lobe	1.992	1.159–3.423			3.874	1.997–7.517	
Others	1.000				1.000		
**Multifocality**			**< 0.001**				**0.006**
Present	2.728	1.751–4.251			3.108	1.378–7.009	
Absent	1.000				1.000		
**Bilaterality**			**0.002**				0.991
Present	2.029	1.287-3.200			1.005	0.429–2.353	
Absent	1.000				1.000		
**Extra-thyroidal extension**			**< 0.001**				**0.022**
Present	2.566	1.592–4.137			1.962	1.104–3.487	
Absent	1.000				1.000		
**Extra-nodal extension**			**< 0.001**				**0.024**
Present	4.333	2.295–8.184			2.227	1.111–4.464	
Absent	1.000				1.000		
**CLNM**			**< 0.001**				**< 0.001**
Present	5.611	3.480–9.045			5.299	2.948–9.527	
Absent	1.000				1.000		

### Distinct risk factors of LLNM according to age

As age played a significant role in LLNM, we further investigated and compared independent predictors of patients of different ages. For patients younger than 30 years old, the univariate analyses revealed that sex(*p* = 0.039), tumor size(*p* = 0.046), tumor location(*p* = 0.047), multifocality(*p* = 0.017), extra-thyroidal extension(*p* = 0.010), extra-nodal extension(*p* = 0.011), and CLNM(*p* = 0.003) were related to LLNM. Further multivariate analyses showed that sex(*p* = 0.033), tumor size(*p* = 0.027), tumor location(*p* = 0.020), and CLNM(*p* = 0.019) were independent predictors for LLNM. Surprisingly, apart from CLNM, which had an 11.011(95%CI: 1.475–82.214) times the OR of LLNM compared to those without CLNM, tumors located in the upper lobe of glands had a 25.780(95% CI: 1.651-402.522) times the OR of LLNM (Table 4). The c-index of the prediction model was 0.811 (95%CI: 0.730–0.892).

**Table 4:** Risk factors of lateral lymph node metastasis of patients younger than 30 years old

	LLNM			Univariate analysis			Multivariate analysis	
Variables	Present(*n* = 100)	Absent(*n* = 20)		OR(95% CI)	*p* value		OR(95% CI)	*p* value
Male	33(33.0%)	2(10.0%)		4.433(0.970-20.251)	**0.039**		6.242(1.163–33.497)	**0.033**
Tumor size ≥ 20 mm	32(32.0%)	2(10.0%)		4.235(0.926–19.367)	**0.046**		7.753(1.259–47.753)	**0.027**
Upper lobe	25(25.0%)	1(5.0%)		6.333(0.806–49.750)	**0.047**		25.780(1.651-402.522)	**0.020**
Multifocality	59(59.0%)	6(30.0%)		3.358(1.191–9.462)	**0.017**		3.293(0.820-13.225)	0.093
Bilaterality	36(36.0%)	6(30.0%)		1.313(0.464–3.713)	0.608			
Extra-thyroidal extension	78(78.0%)	10(50.0%)		6.682(1.309-9.600)	**0.010**		1.989(0.557–7.110)	0.290
Extra-nodal extension	33(33.0%)	1(5.0%)		9.358(1.200-72.958)	**0.011**		5.321(0.598–47.355)	0.134
CLNM	95(95.0%)	15(75.0%)		6.333(1.635–24.526)	**0.003**		11.011(1.475–82.214)	**0.019**

As for patients between 30 and 45 years old, tumor size(*p* = 0.049), multifocality(*p* < 0.001), bilaterality(*p* < 0.001), extra-nodal extension(*p* = 0.015), and CLNM(*p* < 0.001) were found to be related to LLNM. After including all factors above in multivariate analyses, CLNM(*p* = 0.007) was identified as the only predictor of LLNM and showed a 2.990(95% CI: 1.344–6.648) times the OR (Table 5). CLNM had a c-index of 0.626 (95%CI: 0.547–0.705) in predicting LLNM.

**Table 5:** Risk factors of lateral lymph node metastasis of patients between 30 and 45 years old

	LLNM			Univariate analysis			Multivariate analysis	
Variables	Present(*n* = 196)	Absent(*n* = 42)		OR(95% CI)	*p* value		OR(95% CI)	*p* value
Male	71(36.2%)	11(26.2%)		1.601(0.758–3.379)	0.214			
Tumor size ≥ 20 mm	39(19.9%)	3(7.1%)		3.229(0.948–10.999)	**0.049**		2.580(0.719–9.251)	0.146
Upper lobe	59(30.1%)	10(23.8%)		1.378(0.636–2.985)	0.415			
Multifocality	128(65.3%)	13(31.0%)		4.199(2.050–8.603)	**< 0.001**		2.384(0.835–6.810)	0.105
Bilaterality	99(50.5%)	8(19.0%)		4.338(1.911–9.844)	**< 0.001**		1.921(0.582–6.338)	0.284
Extra-thyroidal extension	151(77.0%)	29(69.0%)		1.504(0.722–3.134)	0.274			
Extra-nodal extension	76(38.8%)	8(19.0%)		2.692(1.183–6.124)	**0.015**		1.522(0.621–3.728)	0.358
CLNM	166(84.7%)	25(59.5%)		3.763(1.816–7.797)	**< 0.001**		2.990(1.344–6.648)	**0.007**

For patients over 45 years old, tumor location(*p* = 0.026), extra-thyroidal extension(*p* < 0.001), extra-nodal extension(*p* = 0.001), and CLNM(*p* < 0.001) were significant in univariate analyses. Further multivariate analyses confirmed that tumor location(*p* = 0.013), extra-thyroidal extension(*p* < 0.001), extra-nodal extension(*p* = 0.042), and CLNM(*p* < 0.001) were all independent risk factors to LLNM. And extra-thyroidal extension showed the highest OR of LLNM of 13.005(95%CI: 3.202–11.371) times (Table 6). The c-index of the prediction model was 0.852 (95%CI: 0.781–0.923).

**Table 6:** Risk factors of lateral lymph node metastasis of patients older than 45 years old

	LLNM			Univariate analysis			Multivariate analysis	
Variables	Present(*n* = 100)	Absent(*n* = 41)		OR(95% CI)	*p* value		OR(95% CI)	*p* value
Male	34(33.0%)	8(19.5%)		2.125(0.885–5.104)	0.088			
Tumor size ≥ 20 mm	22(22.0%)	7(17.1%)		1.370(0.535–3.511)	0.511			
Upper lobe	39(39.0%)	8(19.5%)		2.637(1.104–6.299)	**0.026**		3.897(1.336–11.371)	**0.013**
Multifocality	75(75.0%)	24(58.5%)		2.125(0.985–4.584)	0.052			
Bilaterality	63(53.0%)	20(48.8%)		1.788(0.858–3.727)	0.119			
Extra-thyroidal extension	96(96.0%)	27(65.9%)		12.444(3.784–40.922)	**< 0.001**		13.005(3.202–52.822)	**< 0.001**
Extra-nodal extension	35(35.0%)	3(7.3%)		6.821(1.964–23.691)	**0.001**		4.309(1.057–17.569)	**0.042**
CLNM	78(78.0%)	13(31.7%)		7.636(3.396–17.171)	**< 0.001**		8.600(3.217–22.992)	**< 0.001**

## Discussion

Previous studies have reported many risk factors related to LLNM in PTC, including age, sex, tumor size, tumor location, extra-thyroidal invasion, multifocality, and CLNM [18, 19]. As for age, younger patients tend to be more likely to have LLNM than older ones [15–17]. This is similar to our findings. In our study, the figure that demonstrated the trend of the OR of LLNM decreased rapidly as age grew, especially after the specific age threshold. This suggested that a separate prediction system of LLNM should be constructed for patients of different ages to achieve better sensitivity and specificity.

It was generally agreed that younger patients were more likely to have LNM. Still, the exact relationship between the risk of LNM and a decrease in age was shown in one large population research based on the SEER database, which aimed to investigate the impact of age on the risk of LNM; patients were divided into five subgroups by age. The result showed that younger patients had an increased predisposition for LNM [20]. However, the comparison groups were divided according to the cut points of age defined by authors and failed to demonstrate the difference in risk of LNM between groups. We provided a similar result that younger patients were more likely to have LLNM. Apart from that, our division of patients based on age was more accurate and reliable, since the cut points of age were determined based on analyzing the risk of LLNM in each patient subgroup stratified by age in 5-year intervals. The structural breakpoint of the OR fitting curve was identified using the R package, and it found that 30 and 45 were the optimal cut points for the possibility of LLNM.

Many studies have found that CLNM was related to LLNM, and one Meta-analysis showed that the risk of LLNM of CLNM was 7.64 times more than those patients without CLNM [21]. Some researchers suggested that when the number of CLNM > 3, LND should be more actively performed [22, 23]. Additionally, our previous study showed that the risk of occult metastasis in the lateral compartment was much higher for patients with CLNM [24]. Tumor located in the upper pole was another risk factor for LLNM due to an abundant blood supply and direct lymphatic vessels between the upper lobe of the thyroid and lateral neck [25]. Extra-thyroidal extension was an independent predictor for LLNM revealed by several studies as an extension may indicate the tumor’s aggressiveness and lead to metastasis.

For patients with occult LLNM, insufficient treatment may lead to residual tumor or relapse, and secondary operation carries a higher risk of surgical complication and leads a psychological and economic burden to patients. Therefore, our study could be interpreted as an implication for a change in surgical management. In the condition of CLNM being observed before or during surgery, detection of the lateral compartment should be considered. For patients younger than 30 years old, male gender and large tumor size, especially tumor located in the upper lobe, were risk factors that required more attention and appropriate expansion of the extent of surgery when needed. The sufficient surgical extent was more critical for patients older than 45 years old with a poorer prognosis. When finding tumors with extra-thyroidal extension during operation, including gross and minimal, a careful evaluation of potential LLNM was highly recommended.

Despite these findings, we acknowledge that there were still some limitations in this study. First, there is an inherent bias in all single-center and retrospective studies. Second, our sample size was relatively small, especially the group of patients without LLNM, which could reduce the statistical power of this research. Some merits of this study should also be noted. First, the preoperative evaluation of suspicious lymph nodes was evaluated through high-resolution US, which the same experienced experts performed. This avoided bias of diagnosis due to different experienced physicians to the greatest extent. Second, our dissection of levels III and IV was based on the original thyroid incision and did not involve the accessory nerve, and this would not increase the risk of skin numbness and shoulder dysfunction. Third, a group of pathologists described and documented the characteristics of PTC that may be related to LNM.

## Conclusion

This study indicated that the optimal age cut points to stratify the risk of LLNM were 30 and 45 years old. CLNM was a prominent risk factor for further LNM in all patients, and LND should be more actively performed when CLNM was confirmed. We also revealed other distinct risk factors of LLNM in patients with different age stages. Especially for younger patients with tumors in the upper lobe and older patients with extra-thyroidal extension tumors, more aggressive detection of the lateral neck and more intensive surgical treatment should be considered.

## Data Availability

The data that support the findings of this study are available on request from corresponding authors.
